# Itaconate-based nanoparticles induce immunometabolic reprogramming in macrophages and alleviate diet-induced obesity

**DOI:** 10.21203/rs.3.rs-8604693/v1

**Published:** 2026-01-28

**Authors:** Andrea L. Cottingham, Neda Mohaghegh, Sara M. Kolhatkar, Fanny Xu, Shruti Dharmaraj, Jacob R. Shaw, Sanmoy Pathak, Victor Andrade, Yan Shu, Abhinav P. Acharya, Alireza Hassani Najafabadi, Ryan M. Pearson

**Affiliations:** 1. Department of Pharmaceutical Sciences, University of Maryland School of Pharmacy, 20 N. Pine Street, Baltimore, MD 21201, US; 2. Department of Microbiology and Immunology, University of Maryland School of Medicine, 685 W. Baltimore Street, Baltimore, MD 21201, US; 3. Marlene and Stewart Greenebaum Comprehensive Cancer Center, University of Maryland School of Medicine, 22 S. Greene Street, Baltimore, MD 21201, US; 4. Department of Pharmaceutical Sciences, University of Cincinnati James L. Winkle College of Pharmacy, Cincinnati, OH 45221 USA; 5. Terasaki Institute for Biomedical Innovation, Los Angeles, CA 91367 USA; 6. Case Western Reserve University, Cleveland, OH 44106 USA; 7. Co-corresponding authors

**Keywords:** Macrophage polarization, Inflammation, Obesity, Metabolism, Nanoparticle, Adipose tissue, Thermogenesis, Mitochondria

## Abstract

Obesity is driven by chronic adipose tissue inflammation and macrophage dysfunction. Here, we report a cargo-free nanoparticle (NP) platform derived from itaconate-based polyesters (pITA-NPs) that reprograms macrophage immunometabolism and alleviates diet-induced obesity. pITA-NPs rapidly induce CD206 presentation through a translationally independent mechanism and drive a non-canonical M2-like transcriptional and metabolic state distinct from IL-4/STAT6 polarization, while restraining inflammatory activation and macrophage-adipocyte crosstalk to reduce lipid accumulation. Bioenergetic profiling revealed context-dependent metabolic tuning, with pITA-NPs promoting an M2-like metabolic transition in resting macrophages characterized by increased mitochondrial mass and oxidative phosphorylation, while inducing a metabolically restrained, quiescent-like state in M1-like macrophages. In obese mice, subcutaneous pITA-NP treatment suppresses weight gain, reduces adiposity, promotes adipose tissue beiging and brown fat activation, and mitigates systemic inflammatory responses. Together, these findings establish pITA-NPs as an effective immunometabolic nanotherapy that leverages materials-driven macrophage reprogramming and coordinates immune and metabolic regulation to treat obesity.

Obesity is a chronic low-grade inflammatory disease characterized by immune dysregulation and metabolic dysfunction^1,2^. In mammals, adipose tissue exists primarily as white adipose tissue (WAT), which specializes in energy storage and endocrine regulation, and brown adipose tissue (BAT), which is thermogenic and maintains the balance between energy storage and expenditure^3^. Dysregulation of these adipose depots underlies many obesity-associated conditions, including type II diabetes, metabolic dysfunction-associated steatohepatitis, cardiovascular disease, and cancer, contributing to over $100B in annual healthcare costs^4,5^. Current interventions largely focus on lifestyle modification or pharmacological strategies that alter appetite, nutrient absorption, or energy expenditure. Among these, glucagon-like peptide-1 (GLP-1) receptor agonists such as semaglutide have shown remarkable efficacy in promoting weight loss^6,7^. However, their use is frequently constrained by gastrointestinal side effects and concerns regarding long-term tolerability and variability in patient response^8,9^. Mounting evidence highlights that adipose tissue function is tightly regulated by innate immune cells, particularly macrophages, which are among the earliest and most abundant immune cells recruited to adipose tissues during obesity. Macrophage infiltration not only propagates inflammation but also disrupts metabolic function, underscoring the need for therapeutic approaches that address both the immunological and metabolic dimensions of obesity.

Adipose tissue macrophages (ATMs) are key orchestrators of the adipose tissue microenvironment, and their polarization states shape immune function and metabolism^10,11^. In BAT, ATMs predominantly exhibit an anti-inflammatory, M2-like phenotype that supports tissue homeostasis, promotes angiogenesis, and insulin sensitivity. By contrast, obesity triggers an elevated release of chemokines such as MCP-1, which enhances recruitment of circulating monocytes and their subsequent polarization towards a proinflammatory M1-like state. These ATMs also secrete TNFα, IL-6, and IL-1β which interfere with insulin receptor signaling in adipocytes and blunt mitochondrial biogenesis and function^12^. The resulting inflammatory milieu fosters oxidative stress, impairs fatty acid oxidation, and disrupts thermogenic programs in brown and beige adipocytes. Importantly, the balance between resident M2- and M1-like ATMs not only governs inflammatory tone but also determines the metabolic flexibility of adipose tissue. Thus, the convergence of inflammation, ATM polarization, and mitochondrial dysfunction defines a pathological loop in obesity and underscores the therapeutic potential of strategies aimed at simultaneously restoring macrophage homeostasis and mitochondrial metabolism^13^.

Itaconate (ITA) is a metabolite upregulated during proinflammatory macrophage activation that is derived from the conversion of the tricarboxylic acid (TCA) cycle intermediate *cis*-aconitate by aconitate decarboxylase 1 (ACOD1)^14,15^. Although *Acod1* is increased in adipose tissue during inflammation and obesity, this response alone is insufficient to suppress inflammation or maintain homeostasis^16^. ITA exerts immunoregulatory effects by modulating macrophage activation, suppressing cytokine production, and metabolic rewiring^17^. Conflicting reports demonstrate the influence of ITA treatment on macrophage polarization, where a membrane-permeable ITA derivative (4-octyl itaconate; 4OI) inhibited IL-4-induced M2 polarization by targeting JAK1 or enhanced it through Nrf2 activation in microglia and atherosclerosis^18–20^. Polymers of ITA have been shown to exert immunomodulatory activity, while delivery of ITA by liposomes repolarized M1-like macrophages toward an M2-like phenotype through increased CD206 presentation, treating acute liver failure^21–23^. In obesity, daily high-dose oral administration of ITA reduced weight gain and enhanced BAT activation when initiated alongside high-fat diet (HFD) exposure^24^. Moreover, ITA and its isomers have improved insulin signaling and transcriptionally regulated genes involved in gluconeogenesis, fatty acid oxidation, and lipogenesis in HFD mouse models^25^. Collectively, these findings underscore the need to understand the context-dependent immunometabolic effects of ITA, while also developing improved therapeutic strategies to overcome the frequent and high dosing demands required to simultaneously modulate the inflammation and metabolic dysfunction present in obesity.

In this study, we synthesized a library of ITA-based polyesters (pITA) and formulated “cargo-free” nanoparticles (pITA-NPs) to mechanistically investigate their activation-state-dependent effects on macrophage immunometabolism and evaluate their potential to treat established obesity. Treatment with pITA-NPs rapidly induced CD206 presentation on macrophages in a translationally independent manner, while promoting a non-classical M2-like gene expression profile and metabolic rewiring that was dependent on initial polarization state. In transwell co-cultures, pITA-NP-treated inflammatory macrophages reduced lipid accumulation in adipocytes, consistent with the modulation of paracrine signaling. Administration of pITA-NPs to obese mice significantly reduced weight gain and fat mass, accompanied by beiging of WAT, increased BAT, and induction of thermogenic and mitochondrial biogenesis-associated gene expression. Local delivery also elicited systemic effects, lowering circulating pro-inflammatory and elevating anti-inflammatory cytokine levels. Together, these findings establish pITA-NPs as a potent immunometabolic therapy that reprograms systemic and adipose tissue inflammation, promotes BAT activity, and reduces weight gain in obesity.

## pITA-NP treatment rapidly induces macrophage CD206 presentation

**FIG 1.**
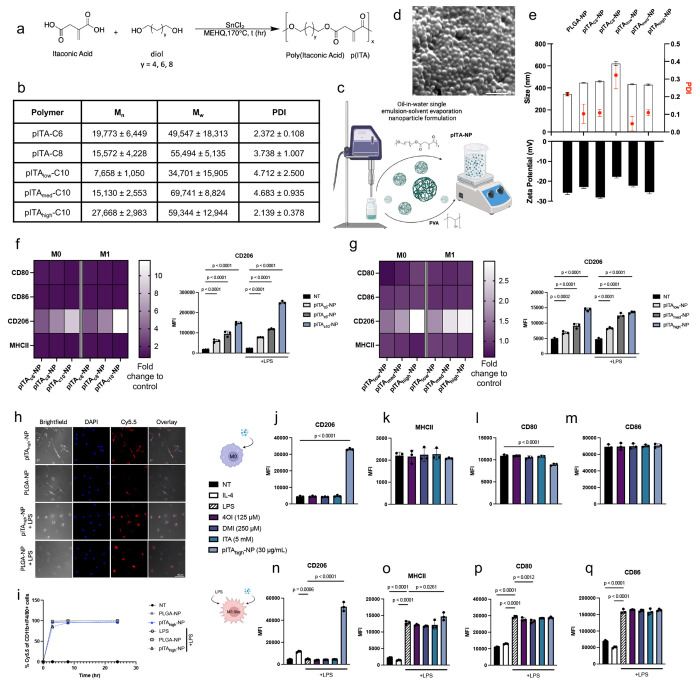
Engineering and immunophenotypic profiling of pITA-NPs. **a**. Polymers were synthesized via a polycondensation reaction using various diols and varying reaction times to alter chain length and molecular weight. **b**. Gel permeation chromatography was used to characterize the polymers. NPs were then formulated via single emulsion (**c**) and characterized using SEM (**d**) and DLS (**e**). Macrophages were polarized to be steady state M0 or pro-inflammatory (100 ng/mL LPS) M1-like overnight, then treated with different chain length NPs (**f**) and different molecular weights (**g**) for 3 hours. Flow cytometry was used to measure the surface expression of CD80, CD86, CD206, and MHCII. Bar graphs are presented as mean MFI values ± SD, where *n* = 3 for each condition. Significance was determined using one-way ANOVA with uncorrected Fisher’s LSD, with a single pooled variance. To assess NP uptake, M0 and M1-like macrophages were treated with 30 μg/mL of Cy5.5-labeled NPs. Confocal images **(h)** taken at 3 hours depict the colocalization of NPs, while flow cytometry shows the % Cy5.5 positive macrophages over time, as measured at 3, 8 and 24 hours **(i)**. pITA_high_-NPs were then compared head-to-head with ITA soluble controls in the M0 and M1-like macrophages, evaluating CD206 (**j**, **n**), MHCII (**k**, **o**), CD80 (**l**,**p**) and CD86 (**m**,**q**) after 3 hours. Bar graphs are presented as mean MFI values ± SD, where *n* = 3 for each condition. Significance was determined using a one-way ANOVA with Dunnett’s multiple comparisons test, with a single pooled variance. Panel **c** created with Biorender.com.

Cell membrane-permeable derivatives of ITA, such as 4OI and dimethyl itaconate (DMI) have been employed to modulate inflammation and metabolism across multiple disease contexts^26–33^. However, these compounds do not recapitulate the biological effects of endogenous ITA and are limited by poor aqueous solubility^34,35^. To overcome these challenges and enable sustained delivery of ITA, we synthesized a set of three ITA-based polyesters (pITA) by bulk polymerization of ITA with 1, 6-hexanediol (C6), 1, 8-octanediol (C8), or 1, 10-decanediol (C10) in the presence of a catalyst and radical inhibitor (Fig 1a). For the C10 polymer, additional variants were synthesized to achieve low, medium, and high molecular weights. The absolute molecular weights and PDIs of pITA were determined using gel permeation chromatography (Fig 1b). Representative ^1^H-NMR and Fourier-transform infrared spectroscopy (FT-IR) confirmed the polymer backbone structure and the presence of the vinylidene alkene associated with ITA (**Supplementary Fig 1**). Nanoparticles (NPs) were then prepared from pITA (pITA-NP) using the single emulsion-solvent evaporation method, with poly(lactic-co-glycolic acid) (PLGA)-NP as a control (Fig 1c). SEM confirmed the spherical morphology of pITA_high_-NP (Fig 1d), and dynamic light scattering (DLS) revealed particle sizes ranging from 350-600 nm with PDIs below 0.35 and zeta potentials between −18 mV to −30 mV (Fig 1e).

To assess composition- and molecular weight-dependent immunomodulatory effects, bone marrow-derived macrophages (BMMØ) were treated with pITA-NPs under resting (M0), prepolarized M1 (LPS)-, or M2 (IL-4)-like conditions. Given the established role of M1 macrophages in driving chronic inflammation and adiposity within WAT, we hypothesized that pITA-NP-mediated metabolic reprogramming could suppress inflammatory activation. Additionally, because adipose tissue harbors resident M0-like macrophages capable of phenotypic plasticity, we reasoned that pITA-NP might bias these cells towards an M2-like state supportive of adipose tissue remodeling and beiging.

Across the three different C-linker formulations, macrophage viability and the presentation of CD206, MHCII, CD80, and CD86 were quantified using flow cytometry (Fig 1f; Supplementary Fig 2,3). Increasing C-linker lengths correlated with greater CD206 surface presentation associated with an M2-like phenotype, with the C10 formulation producing the strongest effect. Further evaluation of the C10 variants revealed a clear molecular weight dependence, with pITA-C10_high_-NP (referred to as: pITA_high_-NP) inducing the most pronounced CD206 presentation across M0, M1-, and M2-like conditions, while only the lowest molecular weight variant modestly reduced viability (Fig 1g; Supplementary Fig 4). In contrast, changes in MHCII, CD80, and CD86 were modest, indicating that pITA-NP design parameters can be rationally tuned to selectively modulate macrophage phenotypes.

To characterize the dynamic effects of pITA_high_-NP activity, particle uptake was confirmed in M0 and M1 macrophages using Cy5.5 labeling, where nearly 100% of cells were positive within 3 hours (Fig 1h,i). CD206 increased rapidly, becoming significant by 30 minutes, peaking at 3 hours, and declining between 8 to 24 hours before returning to baseline by 72 hours (**Supplementary Fig 5**). No changes in CD206 expression were observed with the control treatments, including 4OI or PLGA-NPs. Moreover, CD206 induction by pITA_high_-NP was concentration-dependent, where increasing NP concentration produced greater CD206 presentation (**Supplementary Fig 6**).

Finally, to directly benchmark pITA_high_-NP activity against soluble ITA and established derivatives, macrophages under M0, M1-, and M2-polarizing conditions were treated with free ITA, 4OI, DMI, or pITA_high_-NP for 3 or 24 hours (Fig 1j–q; **Supplementary Fig 7,8**). Across all polarization states and timepoints, only pITA_high_-NP robustly induced CD206 expression, exceeding the modest effects observed with IL-4 stimulation. Taken together, these results demonstrate that pITA_high_-NP exerts immunomodulatory effects that are distinct from soluble ITA and its derivatives, supporting a unique, materials-enabled mechanism of macrophage reprogramming.

## pITA-NP drives context-dependent reprogramming in macrophages

**FIG 2.**
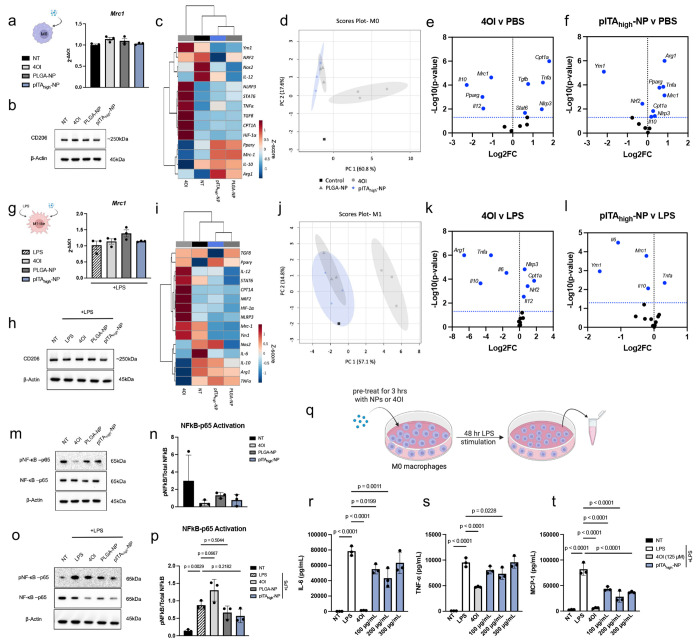
pITA-NPs induce context-dependent transcriptional and inflammatory reprogramming in macrophages. The ability of pITA_high_-NPs to alter transcription and translation was assessed in both M0 and M1-like macrophages (100 ng/mL LPS), respectfully. Cells were treated with 125 μM 4OI, 30 μg/mL PLGA-NP or pITA_high_-NP for 3 hours, after overnight LPS stimulation where noted. RNA was collected for qPCR of *Mrc1* (**a,g**) and proteins were collected for western blots of CD206 expression (**b,h**). Additionally, RNA was collected after 24 hours of treatment, and hierarchical clustering heatmaps (**c,i**), PCA plots (**d,j**), and volcano plots comparing 4OI to no treatment controls (**e,k**), as well as pITA_high_-NP to no treatment controls (**f,l**) were generated. **m,o**. Inflammation after 3 hours was also assessed via protein expression of pNF-κB-p65/total NF-κB-p65 in both M0 and M1- like conditions. Densitometry was used for quantification (**n,p**). Significance was determined using a one-way ANOVA with Dunnett’s multiple comparisons test, with a single pooled variance. **q**. Evaluation of pITA_high_-NPs ability to prevent cytokine release was performed in a concentration manner. M0 macrophages were pretreated for 3 hours with 100-300 μg/mL of pITA_high_-NP or 125 μM 4OI, NP wells washed and media replaced, then stimulated with LPS for 48 hours. Luminex assay was completed to assess for IL-6 (**r**), TNFα (**s**), and MCP-1 (**t**). Bar graphs are presented as mean values ± SD, where *n* = 3 for each condition. Significance was determined using a one-way ANOVA with Dunnett’s multiple comparisons test, with a single pooled variance. Panel **q** created with Biorender.com.

CD206 surface presentation is commonly associated with an alternatively activated M2-like phenotype. However, the rapid induction of CD206 led to the hypothesis that pITA_high_-NP modulates the presentation of CD206 in a translationally independent manner. To confirm this, we performed qRT-PCR of *Mrc1* (the gene encoding CD206) and western blotting of total CD206 protein from M0 and M1 macrophages following treatment with pITA_high_-NP, PLGA-NP, and 4OI for 3 hours (Fig 2a–b,g–h). Notably, under both conditions, no significant increases in *Mrc1* expression nor total CD206 were observed for any groups tested. These results support that pITA_high_-NP treatment likely alters the localization of CD206 through a post-translational mechanism, consistent with the redistribution of intracellular CD206 stores^36,37^.

We next evaluated a focused gene expression panel at 24 hours post-treatment to capture key differences in macrophage activation and metabolic regulation between 4OI, PLGA-NP, and pITA_high_-NP relevant to obesity-associated inflammation (**Supplementary Fig 9, Supplementary Table 1**). Under M0 conditions, hierarchical clustering and principal component analysis (PCA) revealed that pITA_high_-NP and PLGA-NP induced the most similar transcriptional programs, distinct from 4OI, with all treatment groups clustering away from the untreated control (Fig 2c,d). Volcano plots identified differential expression of genes for 4OI and pITA_high_-NP against the PBS control. 4OI promoted a proinflammatory and metabolic activation signature by upregulating *Tnfa*, *Nlrp3*, *Cpt1a*, *Tgfb*, and *Stat6*, while suppressing *Pparγ*, *Mrc1*, *Il12*, and *Il10* (Fig 2e). In contrast, pIT_Ahigh_-NP induced a signature akin to M2-like macrophages with notable increases in *Pparγ*, *Mrc1*, and *Arg1* suggesting a more balanced immunoregulatory response (Fig 2f). Comparison of pITA_high_-NP-treated macrophages with IL-4-induced M2 macrophages revealed distinct gene expression profiles. PCA showed that pITA_high_-NP clustered more closely with PLGA-NP and M0 controls, whereas IL-4 and 4OI treatments each formed separate clusters, suggesting that pITA_high_-NP induces a non-canonical M2-like program that is mechanistically distinct from IL-4/STAT6-driven polarization (**Supplementary Fig 10**).

Under M1 conditions, hierarchal clustering and PCA indicated that the transcriptional responses to pITA_high_-NP and PLGA-NP generated comparable profiles but clearly separated from those driven by 4OI (Fig 2i,j). Notably, 4OI increased proinflammatory and metabolic regulators (*Il12*, *Nlrp3*, *Cpt1a*, and *Nrf2*), while significantly reducing *Arg1*, *Il10*, *Il6*, and *Tnfa*, indicating suppression of inflammatory pathways (Fig 2k). Conversely, the transcriptional impact of pITA_high_-NP on anti-inflammatory *Il10* was less pronounced, while having a divergent response on *Tnfa* and similar reduction of *Il6* when compared to 4OI. A minor, but significant reduction in *Mrc1* expression and a greater than 2-fold reduction in *Ym1*, further highlighting the complex transcriptional effects of pITA_high_-NP on controlling macrophage activation (Fig 2l). Together, these findings highlighted that pITA_high_-NP and PLGA-NP elicit transcriptional programs in M0- and M1-like macrophages that are distinct from those induced by 4OI, reflecting divergent modes of immunomodulatory activity.

Lastly, we examined the impact of 4OI, PLGA-NP, and pITA_high_-NP on NF-κB activation and cytokine production. In M0 macrophages, NF-κB p65 phosphorylation was not significantly altered, whereas in M1 macrophages, LPS-induced p65 phosphorylation by 4OI tended to increase (p = 0.0667) but was slightly decreased by PLGA-NP (p = 0.5044) and pITA_high_-NP (p = 0.2182) (Fig 2m–p). In pre-differentiated M0, M1-, and M2-like macrophages, pITA_high_-NP did not significantly alter basal cytokine secretions (**Supplementary Fig 11**). However, treatment of M0 macrophages with 4OI or pITA_high_-NP prior to LPS stimulation reduced secretion of IL-6, TNFα, and MCP-1, with 4OI exerting the strongest suppression and pITA_high_-NP producing significant but more modest effects (Fig 2q–t). Together with the gene expression data, these results support the divergent, context-dependent effects of 4OI and pITA_high_-NP, where 4OI broadly suppresses proinflammatory responses, while pITA_high_-NP uniquely reprograms macrophages toward a non-classical, CD206-expressing phenotype with more moderate effects on NF-κB and cytokines. These distinct profiles suggest that 4OI more robustly dampens inflammatory signaling, whereas pITA_high_-NP employs a distinct mechanism that may reshape macrophage phenotypes to support anti-inflammatory functions in M0 macrophages, while tempering, but not extinguishing, inflammatory activity in M1 macrophages, a balance that may be advantageous in the metabolically inflamed environment of obesity.

## Divergent mitochondrial and bioenergetic remodeling by pITA-NP and 4OI

**FIG 3.**
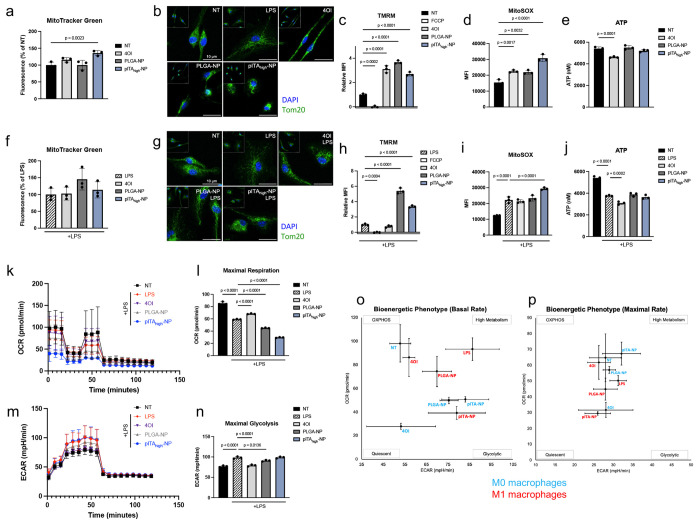
Activation state-selective modulation of mitochondrial function and bioenergetic phenotypes by pITA-NPs. pITA_high_-NPs were evaluated in both M0 and M1-like macrophages on their ability to alter macrophage metabolism. Cells were stimulated with 100 ng/mL LPS overnight to induce an M1-like phenotype, where noted, prior to treatment. Macrophages were treated with 125 μM 4OI, 30 μg/mL of PLGA or pITA_high_-NPs for 24 hours in M0 **(a)** and M1-like **(f)** macrophages. The effects on mitochondrial mass were assessed using MitoTracker Green FM. Additionally, the treatments were evaluated for their effect on mitochondrial potential using flow cytometry with TMRM (**c,h**), the generation of reactive oxygen species with MitoSOX (**d,i**), and their impact on ATP production (**e,j**). Bar graphs are presented as mean values ± SD, where *n* = 3 for each condition. Significance was determined using a one-way ANOVA with Dunnett’s multiple comparisons test, with a single pooled variance. After 3 hours of treatment, the NPs’ ability to alter mitochondrial length, with and without prior LPS stimulation, was observed using confocal microscopy, with TOM20 stained in green and the nucleus labeled with DAPI, shown in blue (**b, g**). Seahorse assays were then performed to measure the changes the treatments have on the oxidative consumption rate (OCR) (**k**), the maximal expiration rate (**l**), as well as the extracellular acidification rate (ECAR) (**m**) and the maximal glycolysis (**n**). Bar graphs are presented as mean values ± SD, where *n* = 3 for each condition. Significance was determined using a one-way ANOVA with Tukey’s multiple comparisons test, with a single pooled variance. With this, the basal rate (**o**) and maximal rate (**p**) of the bioenergetic phenotypes could be determined in both the M0 (blue) and M1-like (red) phenotypes.

Mitochondrial dysfunction is a defining feature of obesity, insulin resistance, and fatty liver disease in both humans and rodents^38^. Thus, we examined how 4OI, PLGA-NP, and pITA_high_-NP alter mitochondrial parameters in M0 and M1 macrophages (Fig 3). In M0 macrophages, pITA_high_-NP enhanced mitochondrial mass (Fig 3a). Further Tom20 staining shows differences in mitochondrial networks that may support divergent activation programs (Fig 3b)^39^. Although all treatments increased mitochondrial membrane potential ΔΨm, the magnitude of change differed, with PLGA-NP producing the strongest effect, followed by 4OI and pITA_high_-NP (Fig 3c). This indicates that each treatment promotes mitochondrial activity, but through different mechanisms or with varying efficiency. ROS measurements further highlighted these differences, while 4OI and PLGA-NP increased mitochondrial superoxide, pITA_high_-NP produced a substantially greater increase, suggesting a stronger oxidative signaling component (Fig 3d). Interestingly, despite these enhancements in mitochondrial activity, only 4OI reduced ATP levels, implying that it may uncouple mitochondrial activity from energy production (Fig 3e). In M1 macrophages, the treatment effects diverged even more sharply. PLGA-NP tended to increase mitochondrial mass and significantly increased membrane potential, indicating an overall strengthening of mitochondrial function (Fig 3f–h). In contrast, 4OI did not elevate ΔΨm or ROS, suggesting that its immunomodulatory activity in inflammatory macrophages is not driven by heightened mitochondrial potential (Fig 3i). Instead, 4OI decreased ATP production, consistent with its capacity to blunt metabolic output and disrupt glycolysis-driven ATP generation in M1 cells (Fig 3j)^40^. Conversely, pITA_high_-NP selectively enhanced mitochondrial potential and ROS production in M1 macrophages, a profile distinct from both 4OI and PLGA-NP suggesting that pITA_high_-NP drives oxidative signaling in inflammatory macrophages, but in a manner that does not increase ATP production, potentially restraining the bioenergetic resources needed to sustain inflammation.

These interpretations were substantiated by Seahorse analysis (Fig 3k–p; **Supplementary Fig 12**). As expected, LPS stimulation reduced OCR and increased ECAR, reflecting the glycolytic shift of inflammatory macrophages (Fig 3k–n). Treatment with 4OI reversed this phenotype by boosting OCR and lowering ECAR, thereby re-establishing OXPHOS.

By contrast, PLGA-NP suppressed both OCR and ECAR, indicative of reduced metabolic flexibility. pITA_high_-NP produced the most significant reduction of OCR, suppressing maximal respiration by 60%, yet left glycolysis intact. This profile suggests that pITA_high_-NP disrupts mitochondrial-driven ATP production without rerouting cells into a glycolytic state, effectively dampening metabolic capacity and limiting the energy available for proinflammatory effector functions. Mapping of bioenergetic phenotypes revealed that these effects were not uniform, but context-dependent (Fig 3o,p). At basal respiration, 4OI-treated M1 macrophages resembled M0 macrophages, both favoring OXPHOS, while pITA_high_-NP in M1 macrophages clustered with glycolytic phenotypes, consistent with impaired mitochondrial respiration. Under maximal stress, pITA_high_-NP produced the largest divergence between polarization states, where in M0 cells an intermediate metabolic profile between OXPHOS and high activity is observed, whereas in M1 macrophages, it shifted cells toward quiescence. In contrast, 4OI promoted an OXPHOS-like phenotype in M1 macrophages but drove quiescence under M0 conditions. These effects underscore pITA_high_-NP does not act as a simple inhibitor of metabolism but rather tunes macrophage bioenergetics differently depending on activation state.

## Macrophage reprogramming by pITA_high_-NP reduces adipocyte lipid accumulation

**FIG 4:**
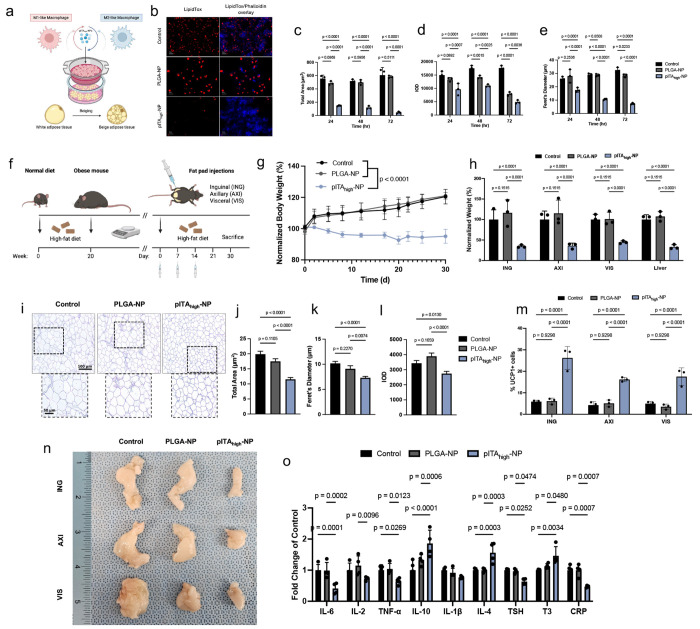
pITA-NPs treatment suppresses adipocyte lipid accumulation and drives robust anti-obesity effects. **a**. Schematic representation of 3T3-L1 adipocyte differentiation into mature adipocytes, then culturing with LPS-stimulated macrophages on a transwell insert for 24 hours. Macrophages were treated with 20 μg/mL NPs for 24, 48 and 72 (shown) hours and stained with LipidTox (red) for LDs and Phalloidin (blue) for actin **(b)** to assess the ability of NPs to alter adipose area **(c)**, integrated optical density (IOD) **(d)** and Fret’s Diameter **(e)** using ImageJ. Bar graphs are presented as mean values ± SD, where *n* = 3 for each condition. Statistical differences were determined by two-way ANOVA with uncorrected Fisher’s LSD test, with p values listed above the compared groups. (**f**) Mice (*n* = 5 per treatment group) were fed high fat diet (HFD) for 20 weeks to induce obesity, then given injections of saline (control), PLGA-NP, or pITA_high_-NP treatment on days 0, 7, and 14 while continuously being fed the HFD. (**g)** Mice were weighed throughout the 30-day treatment period. Bar graphs are presented as mean normalized weight percents ± SD, where *n* = 3 for each condition. Significance was determined using a one-way ANOVA with Tukey’s multiple comparisons test, with a single pooled variance of day 30 weights. The inguinal (ING), axillary (AXI), and visceral (VIS) fat pads were harvested after 30 days and weighted (**h**) and H&E staining was completed on the VIS fat pad (**i**), allowing for total area (**j**), Feret’s diameter (**k**) and integrated optical density (IOD) (**l**) to be calculated. Significance was determined using a one-way ANOVA with Tukey’s multiple comparisons test, with a single pooled variance. The percent UCP1 expression on each of the fat pads was determined using flow cytometry to assess phenotype (**m**). Bar graphs are presented as mean percent positive values ± SD, where *n* = 3 for each condition. Statistical differences were determined by two-way ANOVA with uncorrected Fisher’s LSD test, with p values listed above the compared groups. **n.** Representative images of each of the fat pads. **o**. Additionally, serum cytokines were collected and analyzed for IL-6, IL-2, TNFα, IL-10, IL-1β, IL-4, TSH, T3, and CRP. Statistical differences were determined by ordinary two-way ANOVA with Tukey’s multiple comparisons test, with a single pooled variance. Panels a and f were created using Biorender.com.

Given that macrophage polarization fosters anti-inflammatory niches promoting WAT beiging, and that macrophages are phagocytic and readily internalize NPs, we used a transwell assay to test whether pITA_high_-NP induced macrophage reprogramming alone could indirectly impact adipocyte lipid accumulation (Fig 4a)^41^. Because 4OI induces distinct metabolic effects unrelated to NP treatments, we focused our comparison on pITA_high_-NP versus PLGA-NP, which elicit overlapping yet divergent immunometabolic responses. M1 macrophages were seeded on transwell inserts and treated with pITA_high_- or PLGA-NPs, while fully differentiated 3T3-L1 adipocytes were cultured in the lower chamber for 24, 48, and 72 hours. Adipocyte lipid droplets (LDs) were visualized using LipidTox and Phalloidin staining. Compared to PLGA-NP, pITA_high_-NP-treated macrophages significantly reduced LD number, consistent with a morphological transition of white to beige adipocytes (Fig 4b; **Supplementary Fig 13**). Quantitative analysis revealed several distinct effects of pITA_high_-NP treatment. First, adipocyte total area was significantly reduced at all time points, an effect not observed with PLGA-NP (Fig 4c). Second, the integrated optical density (IOD) of LDs, reflecting lipid accumulation, was markedly decreased in pITA_high_-NP conditions across the 72 hour period, with PLGA-NP also showing reductions at later timepoints (Fig 4d). Finally, the Feret’s diameter (FD) of LDs, a morphological indicator distinguishing unilocular white adipocytes from multilocular brown/beige adipocytes, was significantly reduced by pITA_high_-NP but unchanged by PLGA-NP or control (Fig 4e). Collectively, the reduced area, lower IOD, and smaller FD are indicative of a browning/beiging phenotype, associated with enhanced fatty acid oxidation and thermogenesis. These findings are consistent with pITA_high_-NP-mediated macrophage reprogramming engaging metabolic pathways associated with ITA biology. In this context, altered handling of metabolites such as succinate could contribute changes in macrophage-adipocyte communication, as extracellular succinate has been reported to act as a thermogenic signal in adipocytes through succinate dehydrogenase (SDH)-linked oxidation that promotes thermogenesis and reduces lipid storage^42^. Thus, pITA_high_-NP may promote adipocyte browning through a macrophage-derived, endocrine-like mechanism, providing a possible link between NP-induced macrophage metabolic reprogramming and systemic improvements in adipose tissue function.

## pITA_high_-NP reduces weight gain and promotes adipose tissue beiging in obese mice

Prior to initiation of the therapeutic efficacy studies, we evaluated the toxicity profile of pITA_high_-NPs in naive mice and found no evidence of altered blood chemistry (**Supplementary Fig 14**). The therapeutic efficacy of pITA_high_-NP was next evaluated using a HFD-induced obesity mouse model (Fig 4f). Obese mice received subcutaneous injections of pITA_high_-NP or PLGA-NP into bilateral fat pads (inguinal [ING], axillary [AXI], and visceral [VIS]) on days 0, 7, and 14, while being maintained on a HFD. pITA_high_-NP treatment significantly suppressed weight gain, resulting in an approximate 30% reduction in body mass compared to both untreated and PLGA-NP controls (Fig 4g). Consistent with this effect, tissue mass of ING, AXI, VIS and liver was significantly reduced in the pITA_high_-NP group (Fig 4h). Histological analysis of VIS adipose tissue revealed smaller adipocyte size, decreased LD accumulation, and reduced unilocular morphology, supporting a shift from white-to-beige adipose tissue (Fig 4i–l). Flow cytometry further confirmed enhanced UCP1 expression in ING, VIS, and AXI fat pads of pITA_high_-NP-treated mice, consistent with thermogenic activation (Fig 4m; **Supplementary Fig 15**). Gross comparisons of adipose depots revealed visibly reduced fat accumulation in treated mice (Fig 4n). Additionally, histological analysis of major organs revealed no structural abnormalities, suggesting that pITA_high_-NP was well tolerated without off-target toxicity when given as a therapeutic (**Supplementary Fig 16**). Systemically, pITA_high_-NP decreased circulating proinflammatory cytokines (IL-6, TNFα, and IL-2), while increasing anti-inflammatory cytokines (IL-4 and IL-10), alongside reductions in serum CRP levels (Fig 4o). Thyroid hormone analysis further suggested metabolic regulation, with decreased TSH and elevated T3 levels, consistent with enhanced energy expenditure. These systemic effects were further observed through histological assessment of the fat tissue surrounding the kidney, lung, and spleen, demonstrating reduced adipocyte sizes (**Supplementary Fig 17**). Finally, biodistribution studies showed PLGA-NPs persisted for up to 10 weeks, whereas pITA_high_-NP was mostly cleared by 6 weeks (**Supplementary Fig 18**). Together, these findings demonstrate that pITA_high_-NP not only prevents weight gain and induces beiging of adipose tissue but also elicits systemic anti-inflammatory benefits.

## pITA_high_-NP differentially reprograms adipose tissue and liver gene expression profiles to promote white fat beiging and thermogenesis

**FIG 5.**
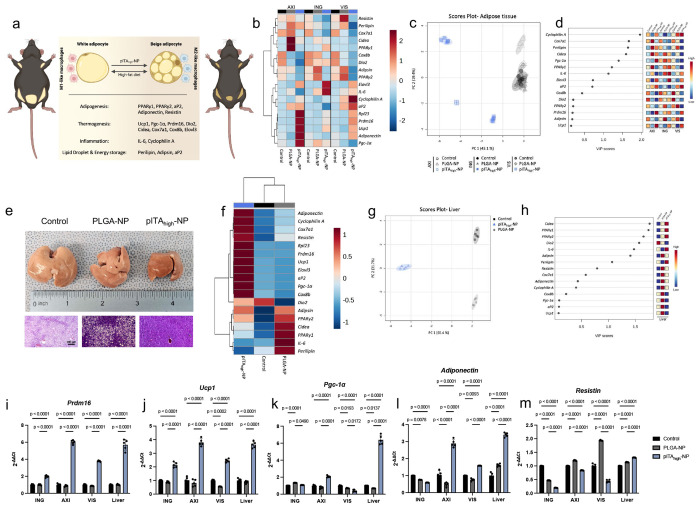
Treatment with pITA_high_-NP induces coordinated transcriptional remodeling across adipose depots and liver. Investigation into key transcripts and transcription factors involved in adipogenesis, thermogenesis, inflammation, LD and energy storage, following NP treatment were explored (**a**). Following qPCR of the inguinal (ING), axillary (AXI), and visceral (VIS) fat pads, hierarchical clustering heatmaps (**b**), PCA plots (**c**), and VIP score plots (**d**) were generated. Livers were isolated and H&E staining performed (**e**), as well as qPCR to generate hierarchical clustering heatmaps (**f**), PCA plots (**g**), and VIP score plots (**h**). Prdm16 (**i**), Ucp1 **(j**), Pgc-1α (**k**), Adiponectin (**l)**, and Resistin (**m**) are just a few of the key transcripts that were investigated in ING, AXI, and VIS fat pads and liver. Bar graphs represent 2^−ΔΔCt^ values, *n* = 5 mice. Error reported as ± SEM. Statistical differences were determined by ordinary two-way ANOVA with Tukey’s multiple comparisons test, with a single pooled variance. Panel **a** was created with Biorender.com.

To directly investigate the molecular changes driving the anti-obesity effects of pITA_high_-NP, we analyzed gene expression profiles in adipose tissues and the liver, focusing on pathways related to adipogenesis, thermogenesis, inflammation, and lipid storage (Fig 5a; **Supplementary Table 2**). Hierarchical clustering and PCA analysis demonstrated that pITA_high_-NP-treated AXI, ING, and VIS samples clustered distinctly from PLGA-NP and untreated controls (Fig 5b,c). Variable importance projection (VIP) scores identified *Cyclophilin*, *Cox7a*, *Perilipin*, *Cidea*, *Pgc-1a*, *Pparg1*, and *Il6* as major contributors to cluster separation (Fig 5d). In the liver, a metabolic organ not directly exposed to NP treatments, we observed markedly reduced hepatic fat accumulation and distinct clustering of pITA_high_-NP-treated mice away from controls (Fig 5e–g). VIP scores showed Cidea, *Pparγ1, Pparγ2, Dio2*, and *Adipsin* were principal drivers of separation (Fig 5h). Importantly, while pITA_high_-NP robustly induced thermogenic and mitochondrial regulators, specifically *Ucp1*, in adipose tissues, it did not strongly upregulate *Pparγ1* or *Pparγ2*. However, both isoforms were significantly increased in the liver (**Supplementary Fig 19**). This divergence highlights potential differences between local adipose remodeling and systemic endocrine-mediated immunometabolic regulation following pITA_high_-NP treatment. Together, these results demonstrate that pITA_high_-NP differentially shapes gene expression signatures in both adipose tissues and the liver, consistent with coordinated regulation of local and systemic energy metabolism and fat remodeling.

At the individual gene level, several BAT- and thermogenesis-associated programs were prominently activated (**Supplementary Fig 19**). *Prdm16*, a master regulator of brown adipocyte identity, was significantly upregulated (Fig 5i). By co-activating *Pparγ* and *Pgc-1a*, *Prdm16* drives the expression of brown fat-selective genes, while suppressing white adipocyte programs, thereby promoting oxidative and thermogenic metabolism. In line with this, *Ucp1* expression was robustly increased (Fig 5j). *Ucp1* uncouples oxidative phosphorylation, dissipating the proton gradient as heat, which is a defining function of BAT. Additional BAT markers were also elevated, including *Elovl3*, which elongates very-long-chain fatty acids to sustain fatty acid oxidation. *Pgc-1a*, a central transcriptional coactivator of mitochondrial biogenesis and oxidative metabolism, was elevated in AXI adipose and liver (Fig 5k), suggesting enhanced metabolic capacity and long-term energy expenditure. Inflammatory and adipokine signaling pathways were also modulated, as *Adiponectin*, an anti-inflammatory and insulin-sensitizing adipokine typically suppressed in obesity, was significantly increased across multiple adipose depots and liver (Fig 5l), while *Resistin*, a pro-inflammatory adipokine linked to insulin resistance, was reduced in adipose tissues (Fig 5m). Interestingly, PLGA-NP treatment elevated *Resistin* in some depots, underscoring the distinct immunomodulatory effects induced by pITA_high_-NP. Collectively, these data show that pITA_high_-NP treatment activates thermogenic and mitochondrial biogenesis programs, suppresses pro-inflammatory adipokine transcription, and remodels adipose tissue toward a BAT-like phenotype. Although upstream regulators were not directly assessed, these coordinated changes are consistent with activation of energy-sensing pathways, such as AMPK. Taken together, the data demonstrate that pITA_high_-NP exerts dual local and systemic effects on immunometabolism, driving WAT-to-BAT conversion, enhancing energy expenditure, and shaping adipose-liver crosstalk.

## Conclusions

In this work, we developed pITA-NPs as a cargo-free immunometabolic therapy to reprogram macrophage polarization and metabolism as a treatment for obesity. pITA-NPs modulated CD206 surface presentation on macrophages through a transcriptionally independent mechanism and elicited context-dependent, non-canonical M2-like transcriptional and metabolic programs in resting M0-like macrophages. When applied to pro-inflammatory M1-like macrophages, pITA-NPs restrained excessive activation and altered macrophage-adipocyte crosstalk in a manner that reduced lipid accumulation, highlighting a potential dual role in restoring macrophage balance within adipose tissue. In obese mice, local administration of pITA-NPs suppressed weight gain and adiposity and promoted thermogenic remodeling through white adipose tissue beiging and brown adipose tissue activation, accompanied by induction of key mitochondrial and thermogenic gene programs. These localized effects were associated with systemic modulation of inflammatory responses, including reduced circulating pro-inflammatory cytokines and increased anti-inflammatory mediators, without evidence of toxicity.

Despite the strength of our findings, certain limitations and future research questions warrant discussion. *In vitro*, the rapid and translationally independent induction of CD206 surface presentation following pITA_high_-NP treatment represents an unexpected observation, and the intracellular trafficking pathways and signaling mechanisms underlying this response remain to be defined. In addition, while our data implicate engagement of mitochondrial and immunometabolic pathways linked to ITA biology, the specific mediators governing macrophage-adipocyte communication require further investigation. *In vivo*, macrophage phenotypes and metabolic states were not directly profiled within adipose tissues. Accordingly, the observed outcomes are interpreted as being consistent with macrophage-mediated mechanisms that are inferred from complementary *in vitro* data. In this context, our data suggest that the anti-obesity effects of pITA_high_-NP *in vivo* are likely driven by reprogramming of resident or recruited M0-like macrophages toward non-canonical M2-like phenotypes, rather than by direct dampening of established M1 macrophages. Although robust adipose and liver remodeling, thermogenic gene induction, and systemic cytokine modulation were observed, whole body metabolic and thermogenic assessments, as well as glucose or insulin tolerance testing, were not performed. Finally, this study focused on local delivery and short-term efficacy, and future work will be required to assess long-term durability, dosing strategies, and translational potential across additional obesity and inflammatory disease models.

Together, these findings position pITA-NPs as a distinct class of metabolite-based immunometabolic nanotherapeutics capable of selectively reprogramming macrophage phenotypes and mitochondrial metabolic activity. By exploiting NP and ITA-mediated immunoregulation rather than conventional payload delivery, this platform introduces an effective strategy for sustained control of obesity and potentially other inflammatory diseases.

## Materials & Methods

### Reagents:

Acid-terminated 50:50 PLGA (~0.17 dL/g inherent viscosity in hexafluoro-2-propanol; approximately MW 4.2 kDa) was purchased from Lactel Absorbable Polymers (Birmingham, AL). Poly(vinyl alcohol) (PVA) 87-90% hydrolyzed (average mol wt 30,000-70,000), chloroform, methanol, itaconic acid, 1,6-hexanediol, 1,8-octanediol, 1,10-decanediol, Tin (II) Chloride, 4-methoxyphenol (MEHQ), d-Chloroform, d-DMSO, LPS, 4-Octyl itaconate, dimethyl itaconate, insulin, dexamethasone, 3-isobutyl-1-methyl xanthine were purchased from MilliporeSigma (Burlington, MA). Trypsin-EDTA, RPMI 1640 Medium supplemented with L-glutamine, Dulbecco’s phosphate-buffered saline (DPBS), penicillin/streptomycin (Pen/Strep), and Dulbecco’s Modified Eagle Medium (DMEM) were obtained from Invitrogen (Waltham, MA). Heat-inactivated fetal bovine serum (FBS) was purchased from VWR (Radnor, PA). ELISA kits for IL-6, IL-1β, TNF-α, IL-10, MCP-1, IL-2 and IL-4 were sourced from BioLegend (San Diego, CA). Mouse Triiodothyronine (T3), TSH, and C-Reactive Protein (CRP) ELISA kits were obtained from MyBioSource (San Diego, CA). Collagenase Type II, LipidTox, and Dispase II were obtained from Fisher Scientific (Waltham, MA). All other antibodies, chemicals, and kits were sourced as noted in the respective methods sections below.

### Polymer synthesis

Itaconate polymers (pITA) were synthesized via polycondensation reactions by melting together itaconic acid (5 mmol, 650.05 mg) and either 1,6-hexanediol (5 mmol, 590.87 mg), 1,8-octanediol (5 mmol, 731.15 mg) or 1,10-decanediol (5 mmol, 871.5 mg) with 10 mol % SnCl_2_ serving as a catalyst in the presence of 0.5 wt % MEHQ as a radical scavenger. Reactions took place at 170°C under argon with a Dean-Stark distillation receiver attached to remove water. After 0.5, 1, and 2 hours, the reaction was stopped, and the polymer was dissolved in 6 mL of chloroform then precipitated dropwise into 300 mL of cold stirring methanol. The polymer was vacuum filtered, then dried under vacuum for 48 hours to remove any residual solvent. As a control to observe the presence of crosslinking via FTIR, a succinate-C10 polymer was synthesized in a similar manner as pITA-C10. Succinic acid (5 mmol, 590.45 mg) and 1,10-decanediol (5 mmol, 871.5 mg) were melted together at 175°C for 2 hours, under argon, with 10 mol% SnCl_2_ serving as a catalyst. Purification was identical to ITA polymers.

The synthesized polymers were characterized using ^1^H-NMR, Fourier-transform infrared spectroscopy (FTIR), and gel permeation chromatography (GPC). Polymer samples for ^1^H-NMR were dissolved at 10 mg/mL in d-chloroform, while monomers were dissolved in DMSO-d6. FTIR samples were generated using the disk method, with a sample concentration of 0.5-2% combined with KBr. Samples for molecular weight analysis via gel permeation chromatography (GPC) were dissolved at 5 mg/mL in chloroform overnight, filtered using a 0.2 μm filter, then injected on Styragel HR4E and HR3 columns in series, at a rate of 1 mL/min using a Waters Acquity HPLC system (Milford, MA). Triple detection allowed for absolute molecular weight determination using a Malvern Panalytical OMNISEC detector (Westborough, MA). Analysis was performed using Malvern OMNISEC v11.41 software.

### Nanoparticle formulation

NPs were formulated using an oil-in-water single emulsion method. Polymers were dissolved at a sample concentration of 50 mg/mL in chloroform overnight (using a 100 mg batch size). Any undissolved material was pelleted and removed by centrifugation. The polymer solution was transferred to a 20 mL scintillation vial, then 10 mL of 1% PVA was slowly added to form two layers. The solution was sonicated using a Cole-Parmer 500 W Ultrasonic Homogenizer (Vernon Hills, IL) on ice using a 1/8^th^ inch tip, for 30 seconds at 40% amplitude. The sonicated solution was immediately poured into a beaker containing 80 mL of stirring 0.5% PVA. The solution stirred overnight. The particle suspension was filtered through a 40-micron cell strainer and subsequently washed three times using 40 mL of cold deionized H_2_O at 12,000 xg for 20 mins. The final NP pellet was resuspended and aliquoted into 2 mL screwcap microcentrifuge tubes containing cryoprotectant (4% w/v sucrose, 3% w/v mannitol). The tubes were then frozen at −80°C and subsequently lyophilized for 2 days. To generate Cy5.5- labeled NPs, Cy5.5-polymer conjugates were first synthesized, then formulated using 0.5% (w/w) conjugate into the various NP formulations as previously described. NPs were characterized DLS (Malvern Panalytical Zetasizer NanoZSP; Worcestershire, UK) and nanoparticle tracking analysis (NTA) (Malvern Panalytical NanoSight NS300; Worcestershire, UK) to determine their size, polydispersity index (PDI) and zeta potential.

### Isolation and generation of primary bone marrow-derived macrophages (BMMØs):

C57BL/6J (5-7 weeks old) mice were purchased from The Jackson Laboratories (Bar Harbor, ME) and housed in a facility at the University of Maryland, Baltimore, under pathogen-free conditions. All mouse handling procedures were approved by the University of Maryland, Baltimore Institutional Animal Care and Use Committee (IACUC). The femurs and tibias from 5-12 week old C57BL/6J mice were isolated and flushed with BMMØ media (RPMI 1640 supplemented with L-glutamine, penicillin (100 units/mL), streptomycin (100 μg/mL), 10% FBS, and 20% L929 (ATCC, Manassas, VA) cell-conditioned media) using a 1 mL syringe and a 25-gauge needle. Red blood cells were lysed using ACK lysis buffer (Quality Biological, Gaithersburg, MD) and the cell suspension was then passed through a 40 μm cell strainer, counted using an EVE^™^ Automated Cell Counter (NanoEntek, Waltham, MA) with trypan blue, then plated at 2 x 10^6^ cells in 10 cm tissue culture treated petri dishes. Cells were incubated in 5% CO_2_ at 37°C and media was changed on days 3, 6, and 8. Experiments were performed using day 7-10 BMMØs.

### Flow cytometry

Cells were stained in accordance with BioLegend protocols for flow cytometry and further optimized in-house. Data was collected using a Cytek Aurora flow cytometer and analysis was performed using FCS Express 7 (De Novo Software, Glendale, CA, USA). Staining with Live/Dead Green Viability/Cytotoxicity (Thermo Fisher Scientific, Waltham, MA) was done prior to FcR blocking with anti-CD16/32 (clone 93) antibody (BioLegend, San Diego, CA). Samples were fixed using fixation buffer (BioLegend, San Diego, CA) after completion of cell staining using the following antibodies: PE/Cyanine7 anti-mouse F4/80 (clone BM8), PE-Fire 640 anti-mouse I-AI-E (MCHII) (clone M5/114.15.2), Pacific Blue anti-mouse/human CD11b (clone M1/70), Brilliant Violet 510 anti-mouse CD80 (clone 16-10A1), APC/Cyanine7 anti-mouse CD86 (clone GL-1) and Brilliant Violet 711 anti-mouse CD206 (MMR) (C068C2) were all purchased from BioLegend (San Diego, CA).

### Macrophage polarization and Flow Cytometry uptake studies

The effect of NPs on macrophage uptake, activation, and the M2 phenotype marker, CD206, was evaluated using unstimulated M0 and pre-established M1 and M2 BMMØs. To induce 3 different polarization states, BMMØs received no stimulation (M0 phenotype) or overnight stimulation of 100 ng/mL LPS (M1-like phenotype) or 10 ng/mL of IL-4 (M2-like phenotype). As an initial screening, M0 MØs were seeded in a 24-well plate at a density of 0.2 x 10^6^ cells/well, stimulated overnight to induce M0, M1 and M2 MØs, and then treated the next day with 30 μg/mL of PLGA, pITA_C6_, pITA_C8_, pITA_low_, pITA_med_, or pITA_high_-NPs for 3 and 24 hours, in the presence of the respective MØ stimulant. Supernatants were saved for IL-6, MCP-1, and TNF-α ELISAs. Flow cytometry was then used to evaluate key polarization markers to identify the extent of NP induced changes, including MHCII, CD86, CD80, and CD206. The time-dependent effects were evaluated by varying the pITA_high_-NP incubation times with both M0 and M1 MØs, with PLGA-NPs serving as a control. Cells were collected and processed at 3, 8 and 24 hours for flow cytometry to evaluate Cy5.5 (NP+) uptake. Additional timepoints (0.5, 1, 3, 8, 24, 72 hours) were assessed to evaluate the dynamics of the phenotypic marker expression using 30 μg/mL non-Cy5.5 labeled NPs in both M0 and M1-like conditions. Concentration effects of pITA_high_-NPs were also evaluated. M1 MØs were treated with 15, 30, and 100 μg/mL of NPs for 3 hours, with both PLGA-NPs and 125 μM 4-OI serving as controls. The extent of phenotypic changes was assessed in both experiments via flow cytometry. All surface marker data is plotted as the median fluorescence intensity (MFI).

### Confocal microscopy uptake study

NP uptake was assessed using flow cytometry and visualized using confocal microscopy. BMMØs were seeded at 0.5 x 10^5^ cells/well in an 8-well glass Millicell EZ chamber slide (MilliporeSigma, Burlington, MA). To induce an M1-like phenotype, cells were pre-treated with 100 ng/mL LPS overnight prior to NP treatment, M0s remained unstimulated. Cells were treated with 30 μg/mL of Cy5.5-labeled NPs for 3 hours, washed twice with PBS, and then fixed with fixation buffer. Fluoroshield with DAPI (MilliporeSigma, Burlington, MA) was used to mount and stain the slides, a coverslip was immediately added. Once dried, slides were sealed and stored at 4°C until images were taken. A Nikon Eclipse Ti-2 confocal microscope (Tokyo, Japan) was used to image slides.

### Prophylactic NP treatment and Luminex

The ability of the NPs to perform prophylactically was also investigated. BMMØs were seeded at 0.2 x 10^6^ cells/well in a 24-well plate, then pre-treated with 125 μM 4OI or 100-300 μg/mL pITA_high_-NPs for 3 hours. After treatment, cells were washed twice with PBS to remove excess NPs and replaced with fresh media containing 100 ng/mL LPS. After 48 hours of LPS stimulation, the supernatants were collected and samples were run on a Luminex panel for TNF-α, IL-6, and MCP-1 following the manufacturer’s protocols. Data was analyzed using the Luminex xPONENT software v4.3 (Thermo Fisher Scientific, Waltham, MA).

### *In vitro* RNA isolation and Quantitative Real-Time PCR

To evaluate NP induced transcriptional changes, cells were seeded at 1 x10^6^ cells per well in a 6-well plate and stimulated where noted with 100 ng/mL LPS overnight to induce an M1-like phenotype. The next day, cells were treated with 30 μg/mL NPs or 125 μM 4OI for 3 or 24 hours, then washed with PBS. Total RNA was extracted using the RNeasy Mini Kit (Qiagen, Hilden, Germany) according to the manufacturer’s instructions. RNA concentration and purity were measured with a NanoDrop One spectrophotometer (Thermo Fisher Scientific, Waltham, MA), and integrity was confirmed via 2% agarose gel electrophoresis. cDNA was synthesized from 1 μg of total RNA using the High-Capacity cDNA Reverse Transcription Kit with RNase Inhibitor (Applied Biosystems, Waltham, MA). Quantitative PCR was performed using PowerUp^™^ SYBR^™^ Green Master Mix (Applied Biosystems, Waltham, MA) on a QuantStudio 5 Real-Time PCR System (Applied Biosystems, Waltham, MA). Primers for target genes (*IL-10, TGF-1, STAT6, CPT1A, Ym1, Arg1, Mrc1, PParγ, Nos2, TNFα, IL-6, IL-12, NRF2, HIF-1α, NRPL3*) and reference genes (*β-Actin, GAPDH*) were obtained from Integrated DNA Technologies (Coralville, IA). Reactions were performed in technical duplicates with standardized primer and cDNA concentrations. Data was analyzed using QuantStudio Design & Analysis Desktop Software (Applied Biosystems, Waltham, MA). Relative gene expression was determined using the 2^−ΔΔCt method. Data are presented on a log_2_ scale, with standard error of the mean (SEM) shown for each group. The primer sequences used for PCR amplification are listed in **Supplementary Table 1**.

### Western blotting

The NP translational alterations were investigated through western blotting. Cells were seeded in 6-well plates at 1 x 10^6^ cells/well, then stimmed overnight with 100 ng/mL LPS to induce an M1-like phenotype where noted. Treatment followed for 3 hours, with 125 μM 4OI and 30 μg/mL NPs. Cells were harvested, lysed with RIPA buffer and sonicated to isolate nuclear proteins. Cell lysates were pelleted via centrifugation, and proteins extracted. Proteins were denatured with SDS page buffer and run on a gel for separation, then transferred to a solid membrane where they were probed for CD206/MRC1 (E6T5J) XP, pNF-κB-p65 (Ser536) (93H1), NF-κB-p65 (D14E12) XP, and β-Actin (D6A8) (Cell Signaling Technologies, Danvers, MA). Enhanced chemiluminescence (ECL) was used for detection and imaging was performed using an iBright 1500 imager (Thermo Fisher, Waltham, MA), with quantification performed using ImageJ (v2.1.0).

### TOM20 staining

Mitochondrial dynamics were evaluated using Tom20, a translocase on the outer mitochondrial membrane, serving as an outer mitochondrial membrane marker to visualize the mitochondrial network. Day 8 BMMØs were seeded at 0.5 x 10^5^ cells/well. The next day, cells were stimulated with 100 ng/mL LPS for 3 hours, then washed and treated with 125 μM 4OI, or 30 μg/mL of PLGA or pITA_high_-NPs for an additional 3 hours. Following treatment, the cells were washed twice with 1X PBS to remove any extra particles and were fixed with fixation buffer (BioLegend, San Diego, CA) according to the manufacturer’s instructions. Cells were then permeabilized using 0.1% Triton X-100 (MilliporeSigma, Burlington, MA) and blocked with 2% bovine serum albumin (MilliporeSigma, Burlington, MA). The cells were then stained with rabbit TOM20 (#42406) (CST, Danvers, MA) for 3 hours at room temperature. Cells were subsequently washed with 1X PBS remove excess primary antibody and then stained with goat-anti rabbit IgG (H+L) Alexa Fluor Plus 488 antibody (#IC1051G) (Invitrogen, Waltham, MA) for 1 hour. These cells were washed, and chambers were removed. Fluoroshield with DAPI (MilliporeSigma, Burlington, MA) was used to mount and stain the slides, and a coverslip was immediately added. Once dried, the slides were placed in 4°C overnight before imaging. Cells were imaged using Nikon Eclipse Ti-2 confocal microscope (Tokyo, Japan) within 2 days.

### MitoSox, TMRM, MitoTracker, and ATP production

Mitochondrial superoxide (MitoSox), mitochondrial membrane potential (TMRM), mitochondrial mass (MitoTracker Green FM) and ATP production were evaluated individually. BMMØs were seeded in 24-well plates at a density of 0.2 x 10^6^ cells/well and stimulated overnight with 100 ng/mL LPS to induce an M1-like phenotype, where indicated. The following day, cells were treated with 30 μg/mL of PLGA or pITA_high_-NPs, or 125 μM 4OI for 24 hours.

#### MitoSox (Mitochondrial Superoxide)

After 24 hours of treatment, cells were collected and stained with Live/Dead Green, stained for F4/80 and CD11b, then incubated with MitoSox Red (Thermo Fisher Scientific, Waltham, MA) for 20 mins at 37°C. The samples were then analyzed in real-time by flow cytometry. Data plotted as mean MFI, ± standard deviation (SD).

#### TMRM (Mitochondrial Membrane Potential)

Following treatment, TMRM was incubated with the cells for 30 minutes. As a control, noted samples were co-treated with 5 μM FCCP with Image-iT^™^ TMRM Reagent (Thermo Fisher Scientific, Waltham, MA) to induce complete depolarization, serving as the zero reference for relative membrane potential calculations. Cells were then harvested and stained with Live/Dead Green and were analyzed live by flow cytometry. Relative potential = (Sample – FCCP)/(NT – FCCP). Data plotted as relative mean MFI, ± SD.

#### MitoTracker Green FM (Mitochondrial Mass)

Following 24 hours of treatment, cells were harvested, stained with Live/Dead Violet, and incubated with MitoTracker Green FM (Thermo Fisher Scientific, Waltham, MA) for 15 minutes at 37 °C. Samples were then analyzed by flow cytometry. MFIs were normalized to controls (untreated or LPS-treated) and expressed as a percentage of control. Mean values plotted as bar graphs, ± SD.

#### ATP Production

Changes in ATP production by NPs were detected using an ATP Detection Assay Kit (Cayman Chemical #700410, Ann Arbor, MI). After 24 hours of treatment, cells were processed and analyzed following the manufacturer’s instructions. In brief, cells were homogenized by pipetting with 1X ATP Detection Sample Buffer and then diluted 20x for the assay. The assay was performed at room temperature, the same day as sample collection.

### Extracellular flux assays

Oxidation consumption rate (OCR) was measured using a Seahorse Extracellular Flux XF-96 analyzer (Seahorse Bioscience, North Billerica, MA). Briefly, cells were seeded at 0.2 x 10^6^ cells/well in Seahorse XF-96 cell culture plates and cultured with and without LPS (100 ng/mL) overnight. The next day, cells were treated with the treatment groups for 24 hours. After 24 hours post-treatment, media was changed to unbuffered XF-DMEM containing 2.5 mM glutamine, 1 mM pyruvate, and 1 mM glucose. Cells were then starved by keeping them in a non-CO_2_ incubator for 1 hour at 37°C. Subsequently, oligomycin (10 μM), 7 Carbonyl cyanide-4 (trifluoromethoxy) phenylhydrazone (FCCP) (11 μM), and antimycin/rotenone (10 μM) were injected into the drug ports of the calibrant plate, which was kept hydrated with XF-Calibrant solution overnight in a non-CO_2_ incubator at 37°C. After the injection of oligomycin, the OCR measured represented the amount of ATP-linked respiration. After the injection of FCCP, the OCR represented the maximal respiratory capacity. Basal respiration was quantified by measuring OCR prior to the injection of oligomycin.

A similar protocol was followed for extracellular acidification rate (ECAR). Media was changed to unbuffered XF-DMEM containing 2.5 mM glutamine and 1 mM pyruvate, following sequential injections of glucose (10 mM), oligomycin (11 μM), and 2-deoxy glucose (100 mM). Following the injection of glucose, the ECAR was a measurement of glycolysis. After the injection of oligomycin, the recorded ECAR represented the maximal glycolytic capacity. Basal glycolysis levels were measured by analyzing ECAR before glucose injections.

All samples were analyzed with 6 technical replicates and analyzed through Seahorse XF Pro Controller software and Wave Pro v10.1.0 (Agilent Technologies).

### Transwell crosstalk mediated NP treated BMMØ and 3T3-L1 cells

NPs’ ability to alter adipocyte differentiation through macrophage crosstalk was investigated using 3T3-L1 cells (ATCC, VA, USA) cultured in a transwell plate with MØs, as previously reported^43^. Briefly, 3T3-L1 cells were cultured at 0.3 x 10^6^ cells/well in 12-well plates and differentiated into mature adipocytes using a differentiation cocktail consisting of 10 μg/mL insulin, 1 μM dexamethasone, and 0.5 mM 3-isobutyl-1-methyl xanthine. LPS-activated macrophages were seeded on top of a 6 μm pore size cell membrane insert, which was then placed over 3T3-L1 cells. After 24 hours of co-culture, 20 μg/mL of PLGA or pITA_high_-NPs were added directly to the macrophages on the membrane inserts to study the impact of MΦ polarization on the differentiation of 3T3-L1 cells into mature adipocytes. The co-cultures were maintained for 24, 48, and 72 hours. At these time points, adipocyte differentiation was assessed by quantifying lipid droplet (LD) accumulation inside the cells using LipidTox (Thermo Fisher Scientific, Waltham, MA and Phalloidin (Invitrogen, Waltham, MA) staining kits. The total area, Feret’s Diameter (FD), and Integrated Optical Density (IOD) were quantified using ImageJ 1.33 software.

### *In vivo* nanoparticle compatibility study

To assess NP toxicity, healthy C57BL/6 mice (*n* = 3 per group) were injected with 200 μL of treatment (saline, PLGA-NP, pITA_high_-NP; 10 mg/mL NP concentration) *via* tail vein injection. After 24 hours, the mice were euthanized and blood was collected by cardiac puncture. Blood chemistry was measured for alanine aminotransferase (ALT), aspartate aminotransferase (AST), creatine, and Blood Urea Nitrogen (BUN) (VRL Animal Health Diagnostics, Gaithersburg, MD).

### HFD mouse model

Mice handling procedures were conducted under the supervision of the IACUC committee of the Lundquist Institute. Male C57BL/6 mice (8-week-old) were obtained from the Jackson Laboratory (Bar Harbor, ME) and were housed in a temperature-controlled room (22 ± 2°C) with a 12-h light/dark cycle. The mice were separated into two groups: one on a regular diet and one on HFD. Obesity was induced in the HFD group by feeding them HFD for 16-20 weeks, while the non-HFD group was fed a standard normal diet (Fisher Scientific, MA, USA). Body weight was measured twice weekly. Once the HFD mice exhibited a 30–35% increase in body weight compared to the standard normal diet mice, they were grouped together based on their weight to achieve uniform weight distribution between treatment groups.

#### Subcutaneous administration

All HFD-induced obese mice were then divided into three groups: (1) PBS negative control, (n=5); (2) PLGA-NP (n=5); and (3) pITA_high_-NP (n=5). Mice received a total of 2 mgs of the assigned formulation, administered as 6 injections distributed across the three fat pads (inguinal (ING), visceral (VIS), and axillary (AXI)). Starting at day 0, mice were weighed every 3-5 days for the duration of the study. On day 30, mice were euthanized using CO_2_ asphyxiation and the adipose tissues with surrounding tissues and vital organs (heart, kidney, liver, spleen, and lung) were harvested to evaluate the potential systemic toxicity and efficacy of the treatments. ATs around the collected organs (kidneys, lungs, spleen) were also collected to visualize and characterize proximal fat accumulation.

### Isolation of mouse BAT adipocytes

ING, VIS, and AXI adipose tissues were dissected and processed to isolate adipocytes. The tissue was minced using a surgical blade and digested in a digestion buffer consisting of 10 mM CaCl_2_, 3.5 mg/mL Dispase II, and 1 mg/ml collagenase II in 1X DPBS for 60 minutes at 37°C while shaking. EDTA (2 mM) was added to stop digestion, and the cells were then filtered using a 100 μm strainer with 1X DPBS (10 mL) containing 3% BSA. Cells were kept in a Falcon tube for 1-2 hours and the suspended cells were subsequently transferred into a 5 ml Eppendorf tube using wide pipette tips. Finally, the cells were filtered through a 70 μm cell strainer, washed with 3-5 mL of PBS containing 3% BSA, and stained using Zombie Violet fixable viability dye (#423113, lot B381369, Biolegend, San Diego, CA) and anti-mouse UCP-1-AF488 (#225490, lot GR3410497, Abcam, MA) for further analysis by flow cytometry (Ze5, BioRad, CA) according to the previously published method^44^.

### Gene expression analysis of HFD mouse model

Total RNA was extracted from ING, VIS, and AXI ATs using Quick-RNA Miniprep Kits (Thomas Scientific, Chadds Ford Township, PA) according to the manufacturer’s protocol. RNA quality and quantity were assessed using a NanoDrop spectrophotometer (Thermo Fisher Scientific, Waltham, MA) and Agilent 2100 Bioanalyzer (Agilent Technologies, Santa Clara, CA). Only samples with RNA integrity number (RIN) > 8 were used for further analysis. cDNA was synthesized from 1 μg of mRNA using the High-Capacity cDNA Reverse Transcription Kit (Thermo Fisher Scientific, Waltham, MA) according to the manufacturer’s protocol. Quantitative real-time polymerase chain reaction (qRT-PCR) was conducted using Fast SYBR Green Master Mix (Thermo Fisher Scientific, Waltham, MA) on the CFX384TM Thermal Cycler (BioRad, Hercules, CA) qRT-PCR system. The quantitative reactions were replicated five times. Relative target gene expression levels were measured using the 2^−ΔΔCt method, with *GAPDH* used as an internal control gene for normalizing the expression data. Data are presented on a log_2_ scale, with standard error of the mean (SEM) shown for each group. The previously published primer sequences used for PCR amplification are listed in **Supplementary Table 2**^43^.

### Plasma cytokine secretion analysis of HFD mouse model

At the end of the *in vivo* study, blood was collected from mice, and serum was separated using BD Microtainer Tubes with Yellow Caps containing Serum Separator Gel, following the manufacturer’s protocol. The collected serum samples were stored at −20°C until further analysis. ELISA assays for IL-6, IL-2, IL-10, TNF-α, IL-1β, IL-4, TSH, T3 and C-reactive protein (CRP) were performed following the specific procedures provided by the respective manufacturers.

### Biocompatibility and histological analyses

Assessment of systemic toxicity and therapeutic efficacy of pITA_high_-NPs was achieved through biocompatibility and histological evaluation. Following euthanasia, ATs with surrounding tissues and vital organs were collected, rinsed with 1X DPBS, and photographed. The ATs and tissues were then dissected and fixed in 10% paraformaldehyde for at least 48 hours. Next, the samples were processed, embedded, sectioned, and stained using hematoxylin and eosin (H&E) according to standard protocols. Stained sections were then visualized under a Keyence BZ-X710 all-in-one fluorescent microscope (Keyence, Itasca, IL). Quantitative analysis of the adipocyte morphology, including the total area of the adipocytes, integrated optical density, and Feret’s Diameter (FD), were quantified using ImageJ 1.33 software to assess the efficacy of the treatments.

### NP Biodistribution in HFD mice

*In vivo* biodistribution of Cy5.5-labeled nanoparticles (Cy5.5-NPs) was evaluated in HFD mice (*n* = 5) using an IVIS Spectrum In Vivo Imaging System (PerkinElmer, USA). Mice were anesthetized with 2% isoflurane and received an intraperitoneal injection of Cy5.5-NPs at a dose of 16 mg/kg body weight, equivalent to one ING injection. Whole-body fluorescence imaging was performed at 0, 2, 6, 8, and 10 weeks post-injection, using a 1 s exposure time, and images were analyzed with Living Image software (version 4.5, PerkinElmer).

### Statistical analysis

All statistical analyses were performed using GraphPad Prism software (version 10.0, GraphPad Software Inc, CA, USA). Data are presented as mean ± standard deviation (SD) unless otherwise stated. One-way and two-way, post-hoc Tukey ANOVA, Dunnett, and Fisher’s analyses performed, each noted in the figure captions, stating the differences between the data. Statistical significance p < 0.05 is stated above the compared groups, and non-significant differences are not shown.

## Supplementary Material

This is a list of supplementary files associated with this preprint. Click to download.

• SupplementalObesityManuscriptFINAL.pdf

## Data Availability

The main data supporting the findings of this study are available in the paper and the Supplementary Information. Source data will be available from the corresponding authors upon reasonable request.
